# Eye-Hand Synergy and Intermittent Behaviors during Target-Directed Tracking with Visual and Non-visual Information

**DOI:** 10.1371/journal.pone.0051417

**Published:** 2012-12-07

**Authors:** Chien-Ting Huang, Ing-Shiou Hwang

**Affiliations:** 1 Institute of Allied Health Sciences, College of Medicine, National Cheng Kung University, Tainan City, Taiwan; 2 Department of Physical Therapy, College of Medicine, National Cheng Kung University, Tainan City, Taiwan; McMaster University, Canada

## Abstract

Visual feedback and non-visual information play different roles in tracking of an external target. This study explored the respective roles of the visual and non-visual information in eleven healthy volunteers who coupled the manual cursor to a rhythmically moving target of 0.5 Hz under three sensorimotor conditions: eye-alone tracking (*EA*), eye-hand tracking with visual feedback of manual outputs (*EH* tracking), and the same tracking without such feedback (*EHM* tracking). Tracking error, kinematic variables, and movement intermittency (saccade and speed pulse) were contrasted among tracking conditions. The results showed that *EHM* tracking exhibited larger pursuit gain, less tracking error, and less movement intermittency for the ocular plant than *EA* tracking. With the vision of manual cursor, *EH* tracking achieved superior tracking congruency of the ocular and manual effectors with smaller movement intermittency than *EHM* tracking, except that the rate precision of manual action was similar for both types of tracking. The present study demonstrated that visibility of manual consequences altered mutual relationships between movement intermittency and tracking error. The speed pulse metrics of manual output were linked to ocular tracking error, and saccade events were time-locked to the positional error of manual tracking during *EH* tracking. In conclusion, peripheral non-visual information is critical to smooth pursuit characteristics and rate control of rhythmic manual tracking. Visual information adds to eye-hand synchrony, underlying improved amplitude control and elaborate error interpretation during oculo-manual tracking.

## Introduction

Oculo-manual tracking relies on multimodal sensory streams to achieve target goals and coordinate ocular and manual effectors [1]–[3]. Non-visual information from efferent copy and proprioceptive inputs is crucial for the oculo-manual plant to calibrate movement consequences in a conjugate manner [2], [4], [5]. Using a self-moved target design (*SMT*), the respective roles of non-visual inputs from manual effectors have been explored by contrasting behavioral consequences of simultaneous eye and manual tracking with those of eye-alone (*EA*) tracking in healthy and deafferented subjects [5]–[7]. *SMT* tracking exhibits a smoother ocular pursuit, higher pursuit velocity, and less pursuit delay than *EA* tracking [6], [8], [9], due to predictive estimates of pursuit through integration of arm kinesthetic information with ocular commands in the cerebellum [3], [10]–[12]. In addition, arm motor command (efferent copy) is keyed to synchronize ocular motion onset, as latency for ocular tracking of the passively-moved arm is similar to that for *EA* tracking [7]. Subserving to visual guidance of manual tracking, target spatial representation is registered with composite eye movements [13], [14] and then transformed into oculo-manual synergy in target tracking [1], [14]. Although much literature is available on visual and non-visual roles in oculo-manual tracking, little attention has been paid to the following issues of interests. First, the respective role of the non-visual inputs could be different for tracking of an externally-driven target. The reason is that expected sensory consequences during tracking of a bodily target take precedence over feedback processes [5], [9], which external tracking of low frequency relies on in order to remedy tracking deviations [2], [15]. Next, direct comparison of behavioral consequences between *SMT* and *EA* tracking [6], [7] may overstate the proprioceptive contribution to eye-hand synergy during oculo-manual tracking, for improvement of manual action during tracking is partially attributed to visual perception of mismatch between target trajectory and manual consequence. The cognitive effects of visual feedback of manual consequences on tracking improvement should be specified separately.

Movement intermittency is inherent in human oculo-manual tracking [16], [17]. Discontinuity of ocular tracking (or corrective saccade) is an online corrective process for bringing the deviated gaze quickly into the vicinity of the target [13], [18], [19]. As retinal gaze errors of velocity or position can be coded separately in saccade generating networks, saccade genesis seemingly pertains to task condition and spatial uncertainty for the behavior goal [18], [20], [21]. Manual intermittent behaviors (or submovements) are manifested with the velocity profiles of goal-directed aiming [22], [23] and rhythmic tracking [24]–[26]. The submovement superimposes kinematical irregularity on the prototype movement with speed pulses of different scales. Akin to corrective saccades, speed pulses rely heavily on visual feedback for fine-tuning the manual trajectory in accordance with accuracy demands [15], [24], [26]. Intriguingly, the movement intermittency of ocular and manual effectors is cross-modulated by feedback information of their counterpart. Proprioceptive inputs from manual effectors suppress saccadic eye movements [27], and on-line visual feedback enhances discontinuities of the manual output during tracking [15]. Cross modulation of movement intermittency supports the reciprocal transfer of sensory and motor information between ocular and manual subsystems [8], subservient to a fundamental element of eye-hand synergy in the execution of oculo-manual tracking. Despite these facts, no previous studies have specified reciprocity of movement intermittency and error correction processes of the counterpart during rhythmic oculo-manual tracking. It still remains unclear how submovement metrics are adjusted to ocular tracking error and whether saccade events are a cross function of manual tracking error. To gain insight into the relative contributions of non-visual and visual information to achieve a target goal, it is valuable to clarify the intermittency-error reciprocity of the oculo-manual plant, since the central nervous system could weigh different feedback sources to regulate intermittent behaviors under different sensorimotor conditions.

The present work was undertaken to re-examine the respective roles of visual and non-visual information on eye-hand synergy for tracking of an externally-driven target. Our specific foci were 1) the relative contributions of non-visual information to ocular tracking, and 2) the effect of the visibility of manual consequences on scaling of movement intermittency and intermittency-error reciprocity. It was hypothesized that non-visual input during oculo-manual tracking was specified for rate control of manual tracking and enhancement of pursuit characteristics (increments of pursuit incidence and velocity gain). Visual feedback of manual consequences improved the amplitude control of manual tracking with unit displacement gain, accompanied by rescaling of speed pulse metrics (amplitude, duration, and frequency). For the visibility of manual consequence, manual movement intermittency was causally linked to ocular tracking error. Ocular movement intermittency was hypothesized to be triggered by different aspects of manual tracking error, since tracking with and without vision of the manual cursor relies preferentially on visual [2], [28] and proprioceptive feedback [2], [29], respectively.

## Materials and Methods

### Ethics Statement

The experiment was approved by the local human experiment and ethics committee (National Cheng Kung University Hospital Institutional Review Board, NCKUH IRB), and written informed consent was obtained from all the participants, conforming to the Declaration of Helsinki.

### Participants

Eleven right-handed young adults (6 females and 5 males, mean age: 25.5±2.0 years) with normal-to-corrected visual acuity participated in this study. None of them had any known neurological disorders that could affect eye-hand coordination.

### Experimental Setup and Procedures

**Figure 1:**
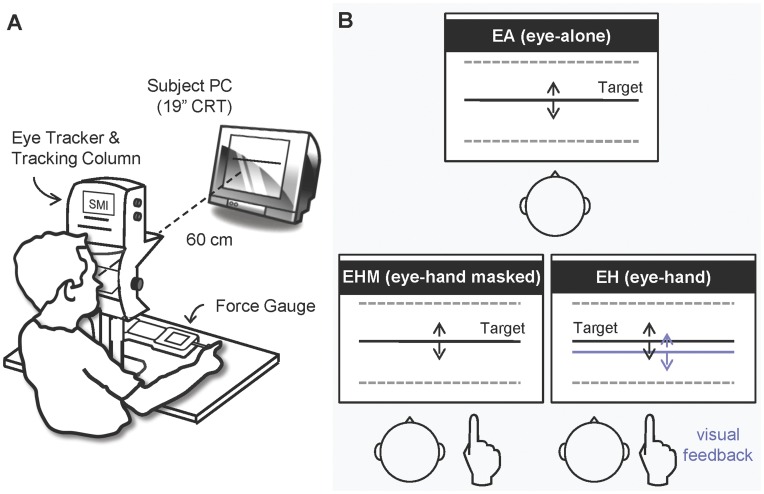
Experimental setup and conditions. (A) A schematic drawing of the experimental setup and the equipment. (B) Diagrammatic representations of the experimental conditions: EA, EHM, and EH tracking. Subjects tracked the target signal (black solid line) visually and/or manually (see the text for details). Timely visual feedback of the manual force output (blue solid line) was available during EH tracking, but was masked during EHM tracking.

Figure 1A illustrates the experimental setup of this study. The subjects were seated 60 cm from a 19″ PC screen (resolution set at 1024×768 pixels) that displayed the target signal at eye level. The head was immobilized on a chin rest and the forehead restraint of an eye-tracking column. The forearm was pronated on the table of the tracking column, and the index finger was pointed forward and hidden from view by the tracking column. The subjects performed a force tracking task by isometrically pressing a multi-function push/pull force gauge (Model: 9810P, AIKOH, Taito-ku, Japan, Capacity: ±100 Nt, Resolution: 0.01 Nt, Accuracy: ±0.2% Full Scale) on the table with the index fingers. A thirteen-point calibration procedure for eye movement was performed before the experiment, according to user guidelines provided by the manufacturer. Vertical eye movements were registered with an infrared illumination video-based eye tracker (iView X Hi-speed 1250, SensoMotoric Instruments GmbH, Germany), and then converted into analog voltage values using a built-in 14-bit D/A board (voltage range: ±10V, Model: PIO-DA4, Taiwan). The eye movement, finger exertion, and target signal were synchronized and digitized at 1.25 kHz using a 16-bit A/D converter (DAQ Card-6036E, National Instruments, Austin, TX, USA) on a LabVIEW platform (National Instruments, Austin, TX, USA).

All the subjects completed tracking tasks under three different conditions in a random order, by visually and/or manually tracking a sinusoidally moving target of 0.5 Hz. The target signal moved vertically in a range of 7.2° of visual angle (i.e., 3.6° above and 3.6° below the eye level on the screen). The maximal velocity was about 11.31 degree/s. In the *eye-alone (EA)* condition, the subjects visually tracked the moving target without any finger action (Fig. 1B). *Eye-hand tracking (EH)* required the subjects to track with the ocular and manual effectors when the visual target and manual cursor were simultaneously displayed on the screen. During *EH* tracking, the subjects coupled the position of the manual cursor to that of the visual target on the screen by scaling the exertion level of the index finger on the force gauge. The minimum and maximum positions of the target trajectory (−3.6°∼3.6° of visual angle) represented force applications of 25 grams and 250 grams, respectively. This resulted in a force-position conversion ratio equal to 0.3125 Nt/degree. The minimal force output during force-tracking was zero gram (−4.4° of visual angle), when the subjects completely released from the force gauge without any force exertion. There would be no negative force output under the current force-tracking setting (i.e., pushing force). Known as kinesthetic tracking, *eye-hand masked tracking (EHM)* was identical to *EH* tracking except that the force output of the index finger (i.e., manual cursor) was not shown on the screen. Practice trials were allowed for each participant to get familiar with the tracking paradigms. All participants were soon able to track the target after 3–5 practice trials, given a vertical display of the level of force exertion on the screen during load-varying isometric abduction of the index finger. In the experiment, each tracking protocol consisted of six trials of 30 seconds with a between-trial interval of 1 minute.

### Data Processing and Statistical Analyses

Eye blinks in the ocular trajectory were detected and replaced with a cubic spline function [30]. If a trial contained more than five eye-blinks (less than 3% of total trials), it was automatically excluded from the subsequent analysis. Vertical eye movements were conditioned with a low-pass filter (cut-off frequency: 20 Hz). The spatial relations among vertical eye movement, finger exertion profile, and target signal were reconstructed in a standardized form of visual angles (°). Subsequent analyses of the rescaled signals included estimations of the following:

#### Tracking performance variables

Ocular and manual tracking errors were defined as root mean square (RMS) values of the mismatch between positional eye movements and finger exertion with respect to the target signal, respectively. Eye-hand trajectory mismatch was the RMS value of the mismatch between positional eye movements and finger exertion. A cross-correlation algorithm was applied to obtain lag-times for ocular and manual tracking with respect to the target signal and lag-time for manual tracking in reference to ocular tracking.

#### Incidences and kinematic properties for composite eye movements

The eye movements during *EHM* and *EH* tracking were principally composed of saccadic and pursuit components. The first and second derivatives of positional eye movements were calculated to obtain velocity and acceleration traces. Saccade was empirically determined by a 750°/s^2^ acceleration threshold and a 15°/s velocity threshold with a minimum dispersion of 0.8° [19], [31]. Maximal velocity values of saccade segments were averaged across trials to obtain the mean peak saccade velocity for each subject. Saccade incidences were obtained by dividing the saccade component duration with the overall duration of a tracking trial, and the remaining portion of eye movements was considered as pursuit incidences. Saccadic variables defined the patterns of ocular movement intermittency. After saccade components were replaced with a cubic spline function [30], pursuit velocity gain was defined as the ratio of RMS velocity of eye movement to that of target velocity signal.

#### Displacement gain, peak frequency, and submovement metrics of finger movements

The displacement gain of finger exertion reflected the amplitude control of manual tracking. It was computed as the ratio of RMS amplitude of finger exertion to that of the target displacement signal. The peak frequency of finger action was derived via a fast Fourier transform to represent the spectral acuity of manual tracking. Greater deviation of the peak frequency from the target rate indicated poorer rate control of the manual tracking. The substructure of the velocity profile of finger action was characterized with speed pulse analysis for continuous rhythmic tracking [24]–[26]. The velocity profile of finger action was obtained by differentiation of displacement trajectory, following 6 Hz low-pass filtering and the removal of the primary movement of the target rate at 0.5 Hz [26]. Duration of speed pulse was the time interval between two successive local minima, and speed pulse amplitude was the difference between the averaged speed value of the two successive local minima and the speed value of an in-between local maximum [24]–[26]. Speed pulse frequency was the number of pulses per second in a tracking trial. All speed pulse variables representing a pattern of manual movement intermittency were determined in the *EH* and *EHM* tracking conditions.

#### Ocular tracking error–finger intermittency relationship

The quantitative analysis was designed to characterize the impact of manual consequences upon adjustments of ocular movement. Linear regression analysis was applied to model the relationships between the scaling properties of speed pulse (amplitude, duration, and frequency) and the amount of ocular positional error for *EH* and *EHM* tracking. A significant regression slope indicated functional linkage between ocular tracking error and speed pulse scaling.

#### Manual tracking error–ocular intermittency relationship

The analysis was designed to evaluate the impact of saccadic response on manual tracking error during *EH* and *EHM* tracking. The mismatches between the finger channel and the target signal in the position profile (positional error) and in the velocity profile (velocity error) were calculated within a set of time window, 200 ms before saccade onset (−200∼0 ms) and 200 ms after the end of the saccade event (0∼200 ms). Regression slopes of the best-fit line for absolute values of manual tracking error before and after saccade were determined. Significant regression coefficients indicated saccade-related adjustments for manual tracking errors. All data analyses were performed off-line using custom-made Matlab scripts (The Mathworks Inc., Natick, MA, USA).

One-way repeated-measures ANOVA and post-hoc paired t-test were used to examine the differences in ocular movement variables (ocular tracking error, ocular tracking delay, and incidences and kinematic properties of composite eye movements) among the three tracking conditions (i.e., *EA*, *EHM*, and *EH*). Paired-t test was used to contrast the manual movement variables (manual tracking error, manual tracking delay, displacement gain, and all speed pulse metrics) between the *EHM* and *EH* conditions. All statistical analyses were completed using the SPSS 17.0 statistical package (SPSS Inc. Armonk, NY, USA) with the significance level set at *P* = 0.05.

## Results

### Tracking Error and Tracking Delay

**Figure 2:**
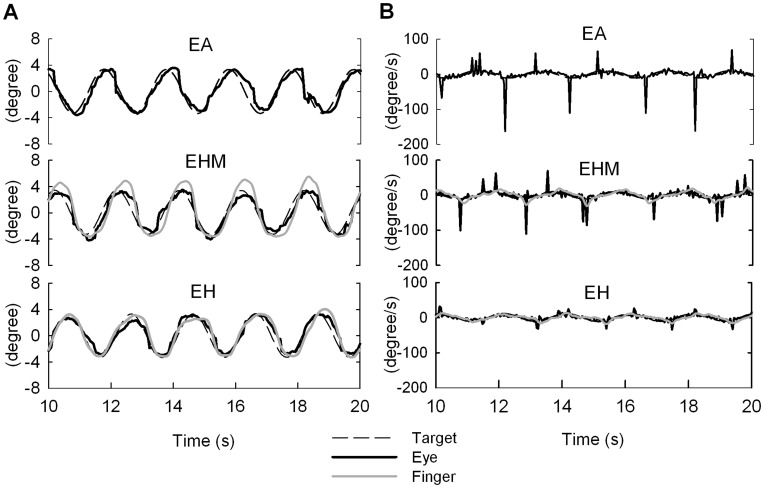
Raw data plots. Central 10 seconds of data of finger action and eye movement from a typical subject under the three tracking conditions. (A) Displacement traces, (B) velocity traces.

**Figure 3:**
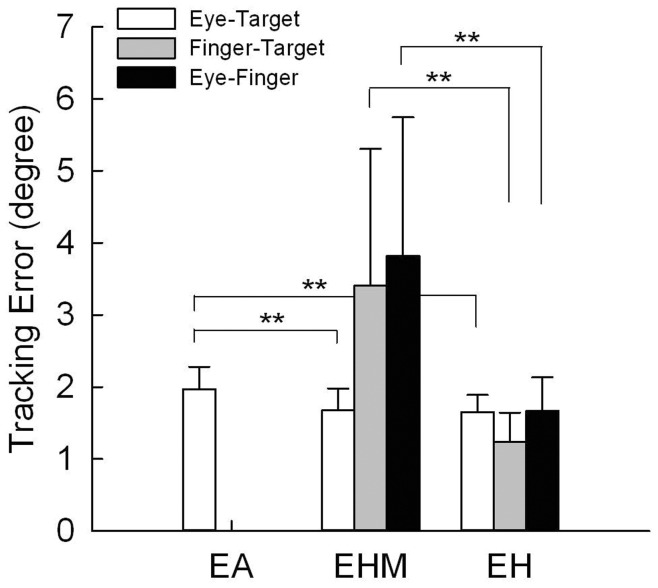
Ocular and manual tracking errors and eye-hand trajectory mismatches for three tracking conditions. (Significant difference, ^**^: *P*<0.01).

Figures 2A and 2B show typical examples of the position and velocity profiles for eye movement, manual output, and target signal for all tracking conditions. Figure 3 shows mean ocular tracking error, manual tracking error, and eye-hand trajectory mismatch in the different tracking conditions. Ocular tracking error varied significantly with tracking conditions (*F*_2, 20_ = 9.44, *P* = 0.001). Post-hoc analysis suggested that ocular tracking error was larger in the *EA* tracking condition than in the *EHM* and *EH* conditions (*P*<0.01), but that it was the same in the *EHM* and *EH* conditions (*P* = 0.719), when manual tracking was a factor. In *EH* tracking, manual tracking error (*t_10_* = 3.62, *P* = 0.005) and eye-hand trajectory mismatch (*t_10_* = 3.65, *P = *0.004) were smaller than in *EHM* tracking. Table 1 summarizes the lag-times for given pairs of eye movement, finger action, and target signal for different tracking conditions. All lag-time variables differed significantly with tracking paradigm (Eye-Target: *F*_2,20_ = 49.54, *P* = 0.000; Finger-Target: *t*_10_ = 4.29, *P* = 0.002; Eye-Finger: *t*_10_ = −2.52, *P* = 0.030). Post-hoc analysis indicated that concurrent finger action and visual knowledge of manual consequence significantly reduced lag-times for ocular (*P*<0.01) and manual tracking (*P*<0.01).

**Table 1:** Lag-times between Eye and Target, Finger and Target, Eye and Finger under different tracking conditions.

	Tracking Condition			
Lag (ms)^1^	EA	EHM	EH	Statistics
Eye-Target	94.44±28.25	44.04±22.88	13.16±10.65	*F*_2,20_ = 49.54, *P* = 0.000 2
Finger-Target	N/A	129.11±52.08	54.21±28.57	*t*_10_ = 4.29, *P* = 0.002
Eye-Finger	N/A	−87.16±66.54	−39.26±17.83	*t*_10_ = −2.52, *P* = 0.03

1 Values were presented as mean ± sd. and all were significantly different from zero (*P*<0.01). Positive values in Eye-Target and Finger-Target mean that eye movement and finger exertion lag behind the target signal, respectively. Negative values in Eye-Finger mean that eye movement leads finger action.

2 Post-hoc for Eye-Target: EA vs.EHM, *P* = 0.000; EHM vs. EH, *P* = 0.003; EA vs. EH, *P* = 0.000.

### Incidences and Kinematic Properties of Composite Eye Movements

**Figure 4:**
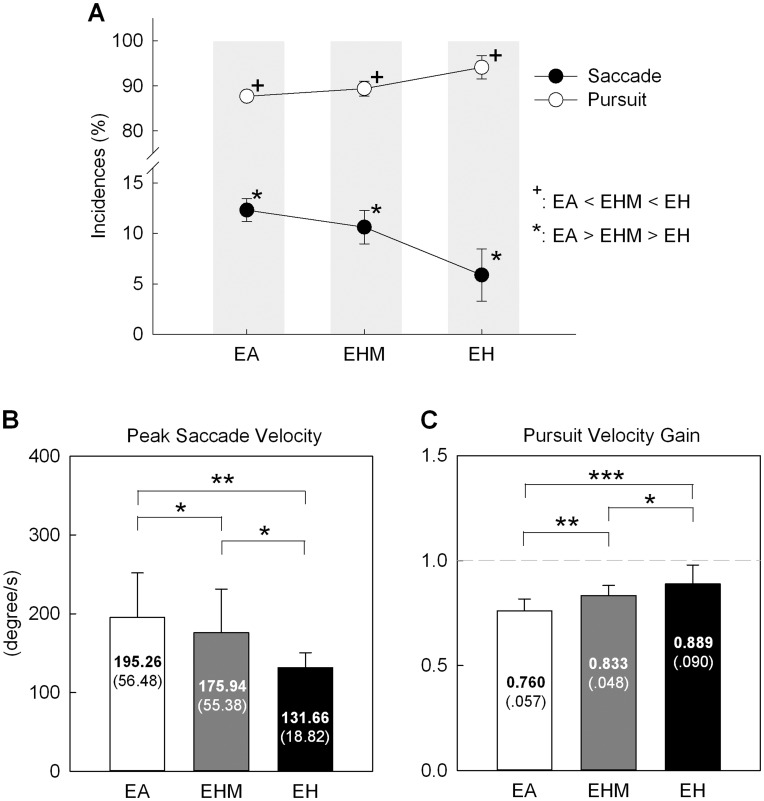
Contrasts of characteristics for composite eye movements under the three tracking conditions. (A) Incidences of saccade and pursuit movements (*: *P*<0.05; ^+^: *P*<0.05), (B) peak saccade velocity, and (C) pursuit velocity gain. (Significant difference, ^*^: *P*<0.05; ^**^: *P*<0.01; ^***^: *P*<0.001).

Figure 4A contrasts the mean incidences of saccadic and pursuit movements under the three tracking conditions. ANOVA statistics revealed that incidences of saccadic (*F*_2, 20_ = 54.20, *P* = 0.000) and pursuit components (*F*_2, 20_ = 54.20, *P* = 0.000) differed among tracking paradigms. Concurrent finger action and visual feedback of manual action suppressed saccade occurrence (*EA*>*EHM> EH*, *P*<0.05), but conversely added to pursuit incidence (*EH* >*EHM*> *EA*, *P*<0.05). Figures 4B and 4C contrast kinematical properties of saccadic and pursuit movements among different tracking conditions. Peak saccade velocity differed with tracking condition (Fig. 4B, *F*_2, 20_ = 9.26, *P* = 0.002), and *EA* and *EH* tracking respectively exhibited the highest and lowest peak saccade velocities among all tracking conditions (*EA* >*EHM* >*EH*, *P*<0.05). Pursuit velocity gain was also a function of shift in tracking paradigms (Fig. 4C, *F*_2, 20_ = 19.10, *P* = 0.000). Contrary to a decreasing trend of peak saccade velocity, pursuit velocity gain increased for tracking with concurrent finger action and visual feedback of the finger action (*EA* <*EHM* <*EH*, *P*<0.05).

### Displacement Gain, Peak Frequency, and Submovement Metrics of Finger Movement

**Figure 5:**
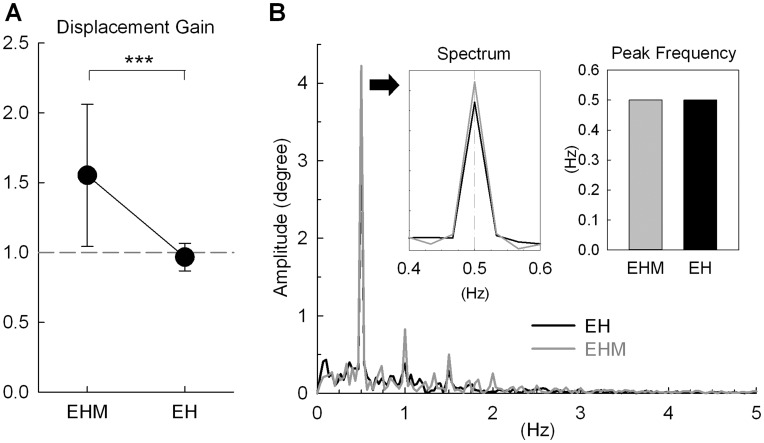
Comparison of manual tracking variables between EH and EHM tracking. (A) Displacement gain; (B) peak frequency, together with a representative amplitude spectrum. (Significant difference, ^***^: *P*<0.001).

**Figure 6:**
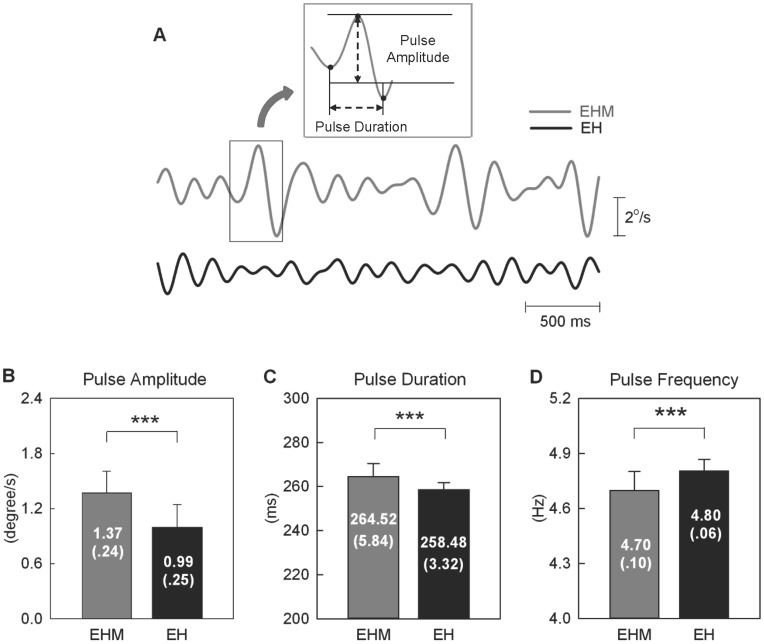
Contrasts of submovement characteristics between EH and EHM tracking. (A) Representative examples of speed pulse traces, with a box showing the measures of pulse amplitude and duration; (B) amplitude, (C) duration, (D) frequency of speed pulses (Significant difference, ^***^: *P*<0.001).

Figure 5A compares the finger displacement gains of *EHM* and *EH* tracking. *EHM* tracking exhibited a greater displacement gain (1.55±0.51) than *EH* tracking (0.97±0.10) (*t_10_* = 3.95, *P* = 0.003). With visual feedback, amplitude of finger movement was almost identical to target amplitude, for the displacement gain during *EH* tracking did not differ from unity (*t_10_* = −1.13, *P* = 0.283 by one-sample *t* test). Figure 5B shows a spectrum plot of finger movement during *EHM* and *EH* tracking; the inset is an enlargement of the spectral peak and a bar chart comparing the average peak frequencies of the two tracking paradigms. All the subjects exhibited similar rate control of the manual tracking, as the spectrum profile peaked consistently at 0.5 Hz for *EHM* and *EH* tracking (Fig. 5). Figure 6A displays representative speed pulse traces of finger movements after removal of a sinusoidal trend from the velocity profiles during *EH* and *EHM* tracking. In *EH* tracking, speed pulses were more frequent (Fig. 6D, *t_10_* = 5.01, *P* = 0.001), of smaller amplitude (Fig. 6B, *t_10_* = 4.80, *P* = 0.001), and of shorter duration (Fig. 6C, *t_10_* = 5.05, *P* = 0.001) than in *EHM* tracking.

### Ocular Tracking Error–finger Intermittency Relationship

**Figure 7:**
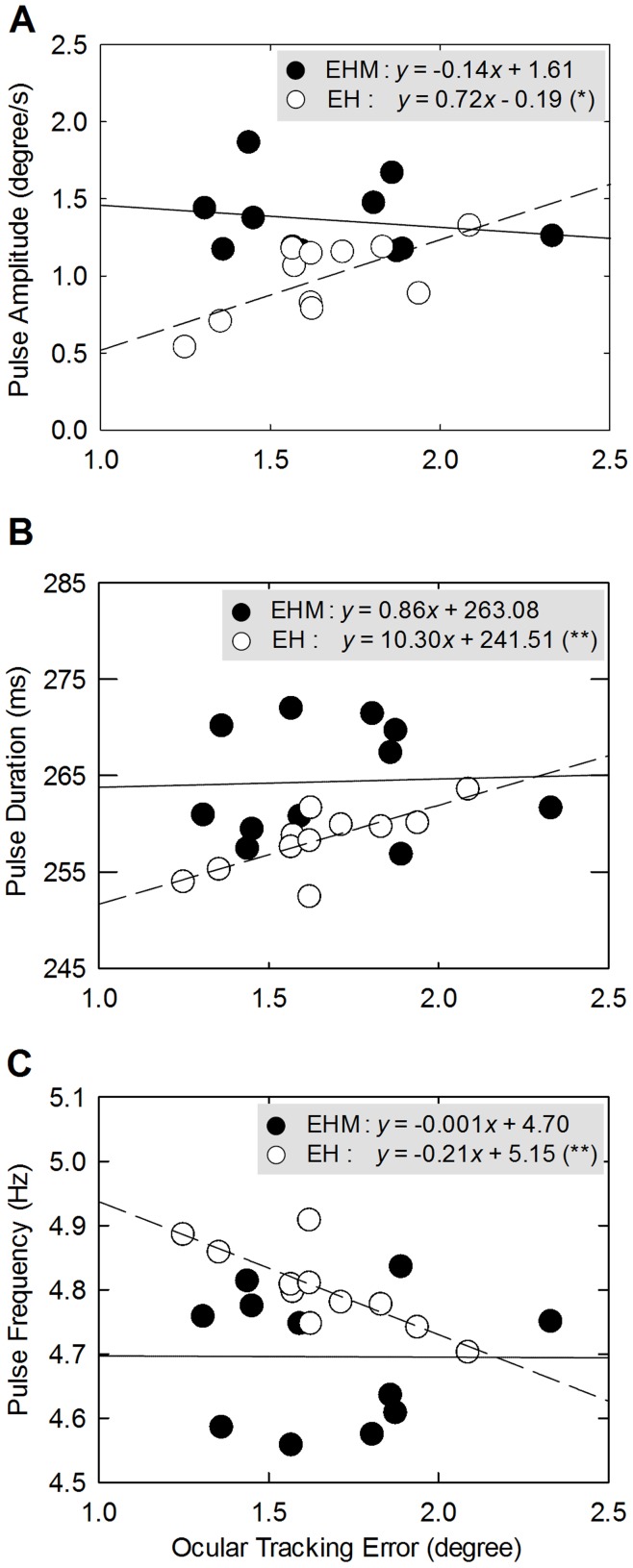
Scatter plots and regression lines of ocular tracking error versus speed pulse metrics. (A) amplitude, (B) duration, and (C) frequency of speed pulses under the EHM (•) and EH (○) conditions. The symbol x in the equations represents ocular tracking error, and the symbol y represents speed pulse metrics in each plot of (A)-(C). (Significant regression slope: ^*^: *P*<0.05, ^**^: *P*<0.01).

Figure 7 displays scatter plots and regression lines of ocular tracking error and speed pulse metrics for *EHM* and *EH* tracking. Ocular tracking error was linearly correlated to speed pulse amplitude during *EH* tracking (*r* = 0.698, *P* = 0.017), but not to speed pulse amplitude during *EHM* tracking (*r* = 0.185, *P* = 0.587) (Fig. 7A). Similarly, ocular tracking error increased in proportion to speed pulse duration during *EH* tracking (*r* = 0.745, *P* = 0.008), but it was independent of speed pulse duration during *EHM* tracking (*r* = 0.045, *P* = 0.896) (Fig. 7B). During *EH* tracking, greater ocular tracking error was associated with less frequent speed pulses (*r* = 0.793, *P* = 0.004), but ocular tracking error was not related to speed pulse frequency during *EHM* tracking (*r* = 0.006, *P* = 0.987) (Fig. 7C).

### Manual Tracing Error–ocular Intermittency Relationship

**Figure 8:**
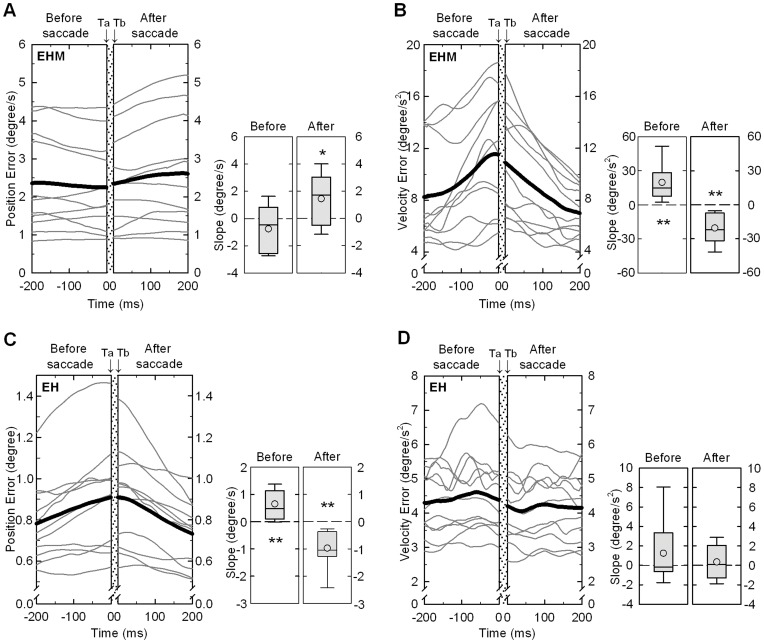
Temporal dispersion and regressive slope for manual positional and velocity errors in the defined window. (A) Positional errors, and (B) velocity errors in the EHM condition; (C) positional errors, and (D) velocity errors in the EH condition. For each plot of (A)–(D), individual (thin line) and mean (thick line) temporal error dispersions are displayed in the left panel. The shadowed area (from time point Ta to Tb) represents an occurrence of a saccade event. The box-plots in the right panel of (A)–(D) show the median (central line), lower and upper quartile (lower and upper lines), and the mean value (circle) for regressive slope of manual tracking errors before and after the saccade event. The lines extending from each end of the box represent the minimum and maximum observations. Asterisks in the box-plots denote significant differences of the regression slope from zero. (^*^: *P*<0.05; ^**^: *P*<0.01; ^**^: *P*<0.01).

Figure 8 contrasts the position/velocity errors of manual tracking within the window (200 ms before and after a saccade event) of *EHM* and *EH* tracking. For *EHM* tracking, the regression slope of positional error of manual tracking before a saccade event (0.75±1.72°/s) was not different from zero (*P* = 0.175), though the regression slope after a saccade event (1.45±1.95°/s) was slightly positive (*P* = 0.034) (Fig. 8A). In contrast, the velocity error of manual tracking for *EHM* tracking was a bell-shaped distribution peaking around the saccade onset (Fig. 8B), with the positive regression slope before saccade (19.70±16.23°/s^2^, *P* = 0.002) and the negative regression slope after saccade (−20.47±13.52°/s^2^, *P* = 0.001). For *EH* tracking, positional error of manual tracking also exhibited a bell-shaped distribution (Fig. 8C). The regression slopes of positional error before (0.65±0.53°/s, *P* = 0.002) and after (−0.98±0.71°/s, *P* = 0.001) saccade were of opposite signs and significantly different from zero. However, the distribution of velocity error was temporally invariant within the defined window (*P*>0.05) (Fig. 8D).

## Discussion

### Concomitant Finger Action and Changes in Ocular Behavior

With an external target design, we noted that mismatch error (Fig. 3) and temporal delay of ocular pursuit were smaller (roughly 50 ms less phase lag) (Table 1) for *EHM* tracking than for *EA* tracking. Non-visual information during *EHM* tracking both increased the pursuit component and suppressed saccadic responses (Figs. 4).

Elaboration of the changes in ocular behaviors is efferent copy for concurrent eye-hand movements and proprioceptive inputs that convey phase information from manual effectors. Primate studies have shown overlapped neural activities of saccadic and manual actions in the frontal eye field, superior colliculus, and cerebellum [11], [32]–[34]. During tracking with non-visual information, proprioceptive inputs could accentuate the phase information transfer in the cerebellum, reinforcing the internal representation of target motion [35] and predictive mode of tracking response [36]. Hence, ocular tracking in the *EHM* condition could be better synchronized with the target signal, leading to infrequent, smaller corrective saccades (Figs. 4A and 4B) and more stable pursuit with a greater velocity gain than in *EA* tracking (Fig. 4C). The alternating modulation of pursuit and saccadic synergies is potentially related to gating mechanisms of omnipause neurons in the brain stem [34], [37]. Considering smooth pursuit with small saccade, *EHM* tracking may involve graded disinhibition of omnipause neurons that suppress the saccade-related ocular motor neurons, accompanied by cooperative tuning of neurons of the rostral superior colliculus [38] and pursuit drive through the cortico-ponto-cerebellar route [34], [37].

Despite the reduction in phase lag of ocular tracking with the aid of manual action, ocular tracking using the external target design exhibited greater phase lag (44 ms in the *EHM* condition) than in previous studies that used a bodily target (5 ms–30 ms) [7]–[9]. The longer phase lag in this study was due to the lower predictability of the external target motion. Koken and Erkelens [27] reported that the complexity of target motion determines the impact of hand movements on ocular tracking. If an unpredictable target is tracked, concurrent manual action has little effect on ocular movement. Hence, a comparably shorter lag time in a bodily target experiment could be explained by proprioceptive inputs [39] and efferent copy signals [40], [41], which enable the subjects to better predict target motion with conscious awareness of action. Another methodological factor that may affect eye-hand synergy, visibility of manual consequence, is discussed in the following section.

### Visibility of Manual Consequence and Changes in Ocular and Manual Behaviors

The contrast in *EHM* and *EH* tracking illustrates the effect of visual feedback of manual consequences upon eye-hand synergy during tracking. It was evident that visibility of manual consequences during *EH* tracking increased the incidence and gain of pursuit movement, but it also further suppressed saccadic responses (Figs. 4A, B, C). In *EH* tracking, manual performance was better than in *EHM* tracking, with less tracking error, smaller phase lag (Fig. 3, Table 1), and better eye-hand synchronization (Table 1). Support for the merits of using visual feedback of manual consequences is that sensory streams are reweighted onto the visual channel [28] so as to alter strategies of visual scanning and error-correction processes during tracking. During *EH* tracking, increases in pursuit gain approaching unity minimized visual tracking error and the need for rapid realignment of the target image on the fovea with corrective saccades. In addition, the visibility of manual consequence enhanced the perception of target motion, which involved intricate oculomotor and attentional networks. The functioning of the inferior parietal lobule is important in establishing maps of extrapersonal space for manual action and target movement [42], [43] through multiple aspects of sensory processing (visual and non-visual) and sensorimotor integration.

Visibility of manual consequences during *EH* tracking modified manual action by rescaling inappropriate gain potentiation in the non-visual condition to a normal unity (Fig. 5A). Without visual feedback of manual consequences, the subjects uniformly perceived generated amplitudes smaller than the actual values during *EHM* tracking because the spatial sensitivity of proprioception was naturally degraded as compared to vision [29]. Strikingly, in pursuit of the sinusoidal target, both *EH* and *EHM* tracking demonstrated manual action with excellent spectral precision (Fig. 5B). This fact implies that the rate of rhythmic oculo-manual tracking could be controlled by non-visual information, despite that amplitude control of manual tracking still remained inadequate. Manual movement intermittency changed with visual feedback of manual consequences, and speed pulses in *EH* tracking were smaller and greater in number than those in *EHM* tracking (Figs. 6B, 6C, and 6D). This observation suggests that visual feedback of manual consequences could modify speed pulse metrics to control the amplitude of manual tracking, as previously reported for continuous tracking tasks [24]–[26] and goal-directed movements [44], [45]. The intermittent servomechanism [15], [16] considers speed pulses as elementary units of an additive accuracy control mechanism, and the limb speed profile is the progressive overlapping and blending of the speed pulses according to accuracy constraints [25], [26], [46]. A smoother movement with a high accuracy demand could allow for more frequent and smaller speed pulses for fine-tuning the movement trajectory. With changes in submovement dynamics, we may well reason that visual feedback of manual consequences improves movement smoothness and spatial accuracy with a reiterated internal feedback process [2], [15], [24]–[26], supplementary to proprioceptive inputs that serve to stabilize temporal acuity and rhythmic control (Fig. 5B) [29], [47].

### Alterations in Interrelationship between Movement Intermittency and Error Detection with Visual Feedback of Manual Consequences

The present study first reported that the availability of visual feedback of manual consequences alters the interrelationship between the accuracy of visual guidance and manual movement intermittency (i.e., speed pulse) (Figs. 7A, B, C). Since ocular tracking error is independent of speed pulse metrics during *EHM* tracking, manual adjustments rely little on the accuracy of visual guidance, for *EHM* tracking makes use of efferent copy and/or proprioceptive inputs as internal cues for manual tracking [31], [48]. In contrast, visually-guided tracking, like *EH* tracking, relies on accurate visual information of target movements to adjust manual outputs with scaled speed pulses. Thus, speed pulse metrics were correlated to ocular tracking error in this study. Poorer ocular tracking congruency demands greater speed pulses for drastic corrections of manual action. When ocular movement was coupled well with the visual target, manual movement was fine-tuned with smaller speed pulses in a more frequent manner. Our observations are in good agreement with the study of Selen et al. [26], who reported that faster visuomotor tracking with greater tracking deviation was associated with a larger speed pulse gain to remedy tracking error quickly.

On the other hand, visibility of manual consequences also affected the reciprocity of manual tracking error and visual movement intermittency (saccadic responses). In the absence of visual feedback of manual consequences, saccadic events during *EHM* tracking responded to the accumulated velocity error of manual tracking, but were insensitive to positional error (Figs. 8A and 8B). During *EH* tracking, the genesis of saccade was conversely a function of positional error, rather than of velocity error of manual action (Figs. 8C and 8D). Our data tended to confirm a switching mechanism for two distributed streams of saccadic control signals (positional error and velocity error) with respect to sensory contexts. Miall et al. [49] showed that monkeys, while tracking a sinusoidal target, can make use of observed positional error and estimated target velocity (efferent copy) to scale the final movement amplitude for each correction attempt. If the positional error of manual tracking exceeds a certain level, a saccadic response is triggered to recalibrate localization information from vision to guide manual action. However, during *EHM* tracking, velocity errors of manual movement triggered saccadic responses (Fig. 8B), probably due to direct use of kinematic information from the manual effectors in the oculomotor vermis and fastigial oculomotor region [11], [50]. These cerebral structures are known to receive Ia excitatory feedback encoding the phase information of manual movement and contraction-induced changes in muscle length during *EHM* tracking [51]. Prsa et al. [50] reported that mossy fiber in the cerebellum discharges coherently with saccade timing for precise directional tuning of ongoing manual movement. In fact, anatomical evidence also supports the operation of two different modes of saccade genesis in position and velocity channels. The middle temporal area appears to process velocity information [52], [53], and the superior colliculus may be responsible for processing position information [20], [21], [54]. Also, parietal-cerebellar circuits are organized to deal with differing error properties before they are transformed into commands for saccade generation during tracking [55].

### Methodological Considerations

The present study investigated eye-hand synergy with vertical target presentation, contrary to most previous studies which examined synchronous ocular-manual behaviors with horizontal target presentation. Although horizontal eye movement is typically greater than vertical eye movement [56], [57], we found that the non-visual effect on ocular movement was largely consistent with previous studies [6], [8], [9], [27]. It was probably because directional asymmetry in ocular behaviors depends on the target kinematics [57], and we used a slow visual target with an acceleration of 50°/s^2^, which could be reliably featured in the normal visual field in both directions [57]. However, the effect of visibility of manual consequences on intermittency-error reciprocity should be generalized to horizontal target presentation with prudence. One potential confounding factor is the different thresholds for perceiving retinal velocity error between vertical and horizontal pursuits [57].

Next, the present study adopted a force-tracking paradigm to investigate eye-hand synergy during oculo-manual tracking. Force tracking differs from positional tracking, as it calls for force-position transformation (0.3125 N/degree) to scale force exertion in terms of visual angles on the screen. Although our subjects could learn to conduct force-tracking after only a few practices, the effects of cognitive effort due to force-position transformation on eye-hand synergy could be further investigated.

Finally, sinusoidal force-tracking in this study often led to a more positional error on manual response in the upward direction, especially around the local maxima of target trajectory during *EHM* tracking. It was not surprising because force-tracking in the upward direction progressively recruited motor units with larger twitch force, known as the size principle [58], [59]. In the absence of visual feedback, force scaling around the local maxima of target trajectory was less accurate but more variable than that around the local minima of target trajectory [60], [61]. Also, the subjects realized a minimal force exertion was necessary in the whole force-tracking process, and they did not allow excessive undershoot around the local minima of target trajectory for releasing from the force gauge without any voluntary effort. Hence, positional error in the upward direction (overshoot) appeared to be more influential to eye-hand synergy than that in the downward direction during the force-tracking maneuver.

### Conclusions

The present study reexamined the respective roles of visual and non-visual inputs during oculo-manual tracking of an externally-driven target. In line with previous studies using a self-moved target design, we found that non-visual inputs and visual guidance contribute to eye-hand synchronization and smoothness of ocular tracking. Non-visual information secures the rate control rather than the amplitude control of manual action which is mainly achieved by scaling of speed pulse variables for visibility of manual consequences. Visual feedback of manual consequences also changes the intermittency-error relationship. Speed pulse variables are correlated to ocular tracking error with the vision of manual cursor, which also switches operation of the visual detection of manual error from the velocity channel during *EHM* tracking to the position channel during *EH* tracking. The present study highlights the fact that movement intermittency is differently scaled with non-visual or visual information, underlying strategic changes in detection and correction of tracking errors.
